# Is Reading Instruction Evidence-Based? Analyzing Teaching Practices Using T-Patterns

**DOI:** 10.3389/fpsyg.2018.00007

**Published:** 2018-02-01

**Authors:** Natalia Suárez, Carmen R. Sánchez, Juan E. Jiménez, M. Teresa Anguera

**Affiliations:** ^1^Departamento de Psicología Evolutiva y de la Educación, University of La Laguna, San Cristóbal de La Laguna, Spain; ^2^Departamento de Psicología Clínica, Psicobiología y Metodología, University of La Laguna, San Cristóbal de La Laguna, Spain; ^3^Faculty of Psychology, Institute of Neurosciences, University of Barcelona, Barcelona, Spain

**Keywords:** teaching practices, reading instruction, T-Patterns, observational methodology, resources, National Reading Panel, components of reading, teaching experience

## Abstract

The main goal of this study was to analyze whether primary teachers use evidence-based reading instruction for primary-grade readers. The study sample consisted of six teachers whose teaching was recorded. The observation instrument used was developed *ad hoc* for this study. The recording instrument used was Match Vision Studio. The data analysis was performed using SAS, GT version 2.0 E, and THEME. The results indicated that the teaching practices used most frequently and for the longest duration were: feedback (i.e., correcting the student when reading); fluency (i.e., individual and group reading, both out loud and silently, with and without intonation); literal or inference comprehension exercises (i.e., summarizing, asking questions); and use of educational resources (i.e., stories, songs, poems). Later, we conducted analyses of T-Patterns that showed the sequence of instruction in detail. We can conclude that <50% of the teaching practices used by the majority of teachers were based on the recommendations of the National Reading Panel (NRP). Only one teacher followed best practices. The same was the case for instructional time spent on the five essential components of reading, with the exception of teacher E., who dedicated 70.31% of class time implementing best practices. Teaching practices (i.e., learners' activities) designed and implemented to exercise and master alphabetic knowledge and phonological awareness skills were used less frequently in the classroom.

## Introduction

There has always considerable interest in exploring how to teach reading and thus bring pupils to appropriate levels of reading proficiency (EACEA/Eurydice, 2011). The National Institute of Child Health and Human Development (2000) identified basic skills that constitute reading competency and the best practices in literacy instruction. Generally speaking, different programs have been developed that propose different ways of targeting the teaching of reading. The direct instruction method (Carnine and Kameenui, 1992; Chard and Jungjohann, 2006; Coyne et al., 2007), based on behavioral theory, is a form of instruction where the teacher is the main axis and works through modeling; this method explicitly uses practices for teaching reading that break the process down into small units, and follows a clear sequence involving repetition and reinforcement. Scaffolding (Temple et al., 2011), based on constructivist principles, consists of having children build their own learning with the help and guidance of their teacher. Psychomotricity, the development of spatial orientation, handedness, and the growing awareness of one's body (Pinker, 2001; Scarborough, 2002; Slavin, 2003), together with respect for one's own pace of learning, are practices based on maturational theory (Fons, 2008). Innatist theory focuses on teaching reading at an early age (Al Otaiba and Fuchs, 2002; De Arcangelo, 2003; Foorman et al., 2003; Fons, 2012; Pascual et al., 2013). Proponents of sociocultural theory promote practices that encourage family, social, cultural, and educational involvement, as all these will play a role in a child's reading development (Purcell-Gates et al., 2002; Fetsco and McClure, 2005; Porta and Ison, 2011; Greenhoot et al., 2014); hence the importance of providing a book-rich environment (Dickinson and Tabors, 2001) that is high in both quality and quantity (Porta, 2008). Finally, the development of phonological awareness through the teaching of sounds is one of the premises of psycholinguistic theory (Pearson, 2001; Rayner et al., 2002; Fletcher-Flinn, 2014). Despite the existence of countless different approaches for teaching reading, the results of international and national tests of basic reading skills [International Association for the Evaluation of Educational Achievement (IEA); Reading Achievement, Progress in International Reading Literacy Study (Mullis et al., 2012; National Assessment of Educational Progress, 2015); Programme for International Student Assessment (Organization for Economic Co-operation and Development, 2012, 2015)]; indicate that it is necessary to improve and promote effective practices.

## Evidence-based practices for teaching reading

Scientific research has demonstrated that the teaching of reading should begin at an early age through teaching practices designed and implemented to exercise and master basic skills that constitute reading competency as defined by the National Reading Panel (2000) (i.e., phonological awareness, alphabetic knowledge, vocabulary, fluency, and comprehension). Phonological awareness means the ability to detect and manipulate sound segments of spoken words (Pufpaff, 2009). Findings on phonological awareness have shown that this is a key skill in the early years of a child's schooling. Many studies have confirmed that this skill is a good predictor of future reading performance (Porta et al., 2010; Suárez et al., 2013; Kjeldsen et al., 2014; Del Campo et al., 2015). Alphabetic knowledge refers to the knowledge of the rules for grapheme-phoneme (G-P) and phoneme-grapheme (P-G) conversion; fluency, which is described as the ability to read texts rapidly and accurately, using appropriate intonation within the reading context; vocabulary, i.e., learning the meaning and use of words in a given context; and comprehension, which refers to a child's ability to reason about, reflect on, and understand what they are reading (Jiménez et al., 2012). Teaching these essential components of reading not only helps children learn to read (National Reading Panel, 2000) it is also helpful for children at risk of exhibiting learning difficulties (Adams, 2001; Foorman and Torgesen, 2001; Tunmer and Arrow, 2013). Numerous recommendations of instructional practices to promote these basic skills have emerged from research findings. The National Reading Panel (2000) developed specific recommendations for activities to teach phonemic awareness. These include isolating, identifying, categorizing, substituting, adding, and deleting phonemes. In the same vein, it has been found that when two or more tasks of segmenting (e.g., dividing a word up into sounds) and deletion (e.g., removing a sound from a given word) are combined, the effect size is much greater.

With respect to alphabetic knowledge, findings have shown that it is better to combine the teaching of sounds with that of the printed letter (Ehri et al., 2001; Stevenson, 2004; Caravolas et al., 2005; Hatcher et al., 2006). It has been shown that the most effective of all the programs using different phonics methods to teach this skill for teaching reading are those that are: synthetic (converting letters to sounds, mixing sounds to form words), analytic (identifying words and their sounds), spelling-based (transforming sounds into letters), contextual (using sound-letter correspondence and finding unknown words in a text), and analogical (using parts of written words to find new ones) (National Reading Panel, 2000). In addition, it's important to note that using a systematic instructional sequence (i.e., easier to more complex and most common letters and letter patterns first) providing ample opportunities for practice and employing evidence-based methods of phonics instruction results in better student outcomes (Armbruster et al., 2001).

Fluency is another skill that predicts reading success. Teachers should teach their pupils to read texts accurately, quickly, and effortlessly, using the correct pronunciation (Nichols et al., 2008), and rapidly, precisely, and with the appropriate intonation (Allington, 1983). It has been shown that guided oral instruction, the use of tutoring, and the involvement of the child's immediate environment have a positive influence on rapid reading (National Reading Panel, 2000). Consolidating this skill also contributes to improving comprehension, as the pupil can free up more cognitive resources for understanding a text (National Institute of Child Health and Human Development, 2000; Hirsch, 2007). Teachers need to use activities focused on: repeated reading of the same text (Rasinski, 2003), independent reading of carefully selected text (Allington, 2000), or practicing expression (Schwanenflugel and Benjamin, 2012), and repeated oral reading with feedback (Armbruster et al., 2001).

Teaching vocabulary also has a direct influence on reading comprehension and vice versa (Perfetti et al., 2005; Hirsch, 2007; Strasser et al., 2013). This skill should be taught early on, and it should focus on the use of strategies such as the use of new technologies, the indirect method, and repeated exposure to words and their meanings (Joshi, 2005; Perfetti et al., 2005; Hirst, 2007; Strasser et al., 2013). Instruction should include multiple exposures to a word, careful selection of words, deepening the meaning of the words, connecting familiar and new words and teaching compound or familiar words (Lane, 2014).

As for comprehension, defined as the skill in which intentional thinking is developed, whereby the meaning of words is constructed through interaction between the text and the reader (Durkin, 1993), a number of practices have proven effective, such as: monitoring comprehension, cooperative learning, the use of graphic and semantic organizers, the use of question-and-answer formats, generating questions, recognizing story structure, and summarizing. In addition, teachers need to help children: activate their prior knowledge, provide ample opportunities to use comprehension strategies (i.e., lower, summarize), read and work with different types of texts (i.e., narratives, expository), use questions to facilitate discussion (Shanahan et al., 2010).

In sum, teachers need to incorporate activities aimed at helping children to discover the sounds of phonemes, associating sounds with the corresponding graphic symbols, creating a link between readings of texts or stories, working with previous knowledge and lexicon, this will help the development of skills such as: phonological awareness, alphabetic knowledge, fluency, comprehension and vocabulary (National Reading Panel, 2000). It has been said that it is important not only that teachers be aware of and understand these components, but also that they know how to work with them to contribute to reading success (Cunningham et al., 2009; Joshi et al., 2009; Kaiser et al., 2009; Podhajski et al., 2009). We must first find out how teachers evaluated actually teach reading, and establish whether their teaching practices are based on the recommendations of scientific research; this is the main aim of the present study.

## Materials and methods

We employed systematic observation, which is widely used in a range of contexts (Castañer et al., 2013, 2016), as it fulfills the basic requirements proposed by Anguera (1979, 2003): habitual behavior, natural context, and perceptivity. These conditions are all guaranteed in the events tracked in our study. The choice of methodology is also justified, as we used an *ad hoc* observation instrument to record, analyze, and interpret how teachers of the sample teach reading.

The observational design can be classified as Nomothetic/Follow-up/Multidimensional (N/F/M) (Blanco-Villaseñor et al., 2003; Sánchez-Algarra and Anguera, 2013; Portell et al., 2015), where *nomothetic* refers to the observation of various different teachers; *follow-up* refers to recording the behaviors or situations that arise over a period of time; and *multidimensional* refers to the fact that more than one dimension of the participant's response is taken into account. We carried out non-participatory observation of teachers on the island of Tenerife (Canary Islands, Spain), in the classroom context, while they were teaching their pupils how to read. Observation was active, governed by scientific criteria, characterized by total perceptibility, and performed by direct observation of the film shot.

### Participants

Our study involved six teachers aged 25 to 50, with 3 to 25 years of teaching experience. Each of them interests us as a case study, individually, and without any pretension of generalizing the results.

The teachers were employed at different preschools and elementary schools on the island of Tenerife. Two of these schools were in a suburban area, one was in a rural area and one was in an urban area. The selection criteria essentially involved ensuring the participants were teachers of language arts and that they spent an average of 1 hour each day on teaching reading.

### Materials

The classroom sessions were recorded using four digital video cameras, four stands and two recorders. Both hardware (two complete computer workstations and two pairs of headphones) and software (Windows Movie Maker by Microsoft, for video editing) were used to observe the teachers' behavior. For recording the data, we used Match Vision 3.0 (Perea et al., 2006). The data quality analysis was done using Generalizability Study (GT) version 2.0.E (Ysewijn, 1996) and the (SAS Institute Inc, 1999) 9.1 statistical package. THEME (Magnusson, 1988) was used to analyze the teachers' behavioral patterns.

The observation of a natural context requires the use of an observation instrument. The observation tool used here was *ad hoc* and combines a field format and systems of categories. The field format is formed by the dimensions of the instrument, and a system of categories has been constructed from each one of them.

This instrument was created using the information obtained from the reality observed, and the dimensions are based on innatist, maturational, behaviorist, sociocultural, corrective, repetitive, and psycholinguistic theories (see Suárez et al., 2013; Jiménez et al., 2014). Systems of categories are characterized by their high degree of structure and their adaptation to the previously defined research question (Anguera, 2003). They also respect the assumptions of mutual exclusivity (e.g., a single behavior cannot be associated with two categories) and exhaustiveness (e.g., a category system covers all possible behaviors ascribed to it). This instrument covers the practices carried out by teachers when teaching reading, and is made up of 14 dimensions. Each criterion has allowed the construction of an exhaustive and mutually exclusive category system.

Below is a presentation of the instrument used in this study (see Table 1). The acronyms shown in the following table (which reflect the wording of the categories in Spanish) were used to record the behavior in the Match Vision Studio program (Perea et al., 2006).

**Table 1:** Observation instrument of practices used for teaching reading.

**Dimensions**	**Categories**	**Acronyms (in Spanish)**
Alphabetic knowledge activities	Teaching sounds	ES
	Teaching letter names	ENL
	Teaching the rule (with graphic symbol)	ERAG
	Teaching the rule (without graphic symbol)	ERSAG
	Rhyming: linking words that rhyme (words can be written in any format: on the board, in notebooks, etc.)	RP
Phonological awareness activities	Stimulating children to become aware of sound (without visual aid)	ECSSV
	Saying words that start with or contain a given sound	DPESD
	Dividing words into syllables	SPS
	Playing “I spy”	JVV
	Separating words in a sentence (clapping or counting)	SPF
	Building words through sounds	CPS
	Rhyming: saying words with the same endings (without graphical symbol as visual aid)	RM
	Saying a sentence with a given words or sound	DFSP
	Alphabet cards	TA
Use of resources	Using made-up stories	HCI
	Songs	CC
	Poetry	PES
	Written material from different sources (menu, children's letters)	EDFMC
	Using written texts or stories	UTCE
	Getting children involved (correcting errors, helping each other, play-acting or role-playing)	HPCAD
	Riddles	ADZ
	Metaphors	UM
	Stories with a moral	MRA
Comprehension previous knowledge activities	Using words the children say	UPN
	Asking children to talk about their experiences	LPNEV
	Reviewing content covered in the past	REPA
Reinforcement	Positive (verbal or tangible)	P(VM)
	Negative (verbal)	NE-VE
Feedback	Correcting reading (mispronunciation, getting lost, skipping a line)	CLPDS
	Pointing out where child went wrong	SDSE
	Offering examples	PEJP
	Alerting child to an error (“no, that's not right”)	AA-NO
	Review and correction of children's work	RCTN
	Asking questions	PG
	The teacher writes the word or sentence wrong	PEMLP
	Repeating the reading	RL
Modeling	The teacher shows the steps to be taken, when the child makes a mistake or to remind the children of content	PEPEQ
Fluency activities	Individual reading (out loud)	LI-VA
	Individual reading (out loud and rapidly)	LIVAR
	Individual reading (silently)	LISIL
	Group reading (out loud)	LG-VA
	Group reading (out loud and rapidly)	LGVAR
	Group reading (silently)	LGSIL
	Fluent reading (rapid, accurate and precise)	FLUI
	Rapid reading (the teachers says “faster”)	LR
	Individual reading with intonation	LEFI
	Group reading with intonation	LEFG
	Individual reading without intonation	LSEFI
	Group reading without intonation	LSEFG
Guided oral instruction	Teaching the steps to be followed (saying them)	EDPS
	Instruction on how to intone sentence or text	IEF
Homework	Doing different activities at home	RVAC
	Reading at home	LECS
Reading and writing activities	Read the word and then write it	LPLE
	Read the sentence and then write it	LFLE
	Writing and reading a sentence	EFL
	Writing and reading a word	EPL
	Dictation of letters, words or sentences	DICPF
	Copying letters, words or sentences	CLPF
	Completing activities or exercises	CA
Psychomotricity activities	Spatial orientation (up, down, left, right, in, out)	OE-AB
	Temporal orientation (yesterday, tomorrow, next year, in a month's time)	OT-AM
	Rhythm (rhythmic sequences)	R-SR
	Body awareness (knowing parts of the body)	EC-CP
Literal or inference comprehension activities	Teaching the parts of a book	EPLB
	Relating illustrations to text	RITEX
	Using news and current events	UNRE
	Using exercises from the book to work on comprehension	REL
	Using questions to check for comprehension of a text	UPC
	Summarizing a text	RTEX
Vocabulary activities	Teaching the meaning of words	ESP
	Using the dictionary	UD

The dimensions refer to whether the teacher carried out teaching practices based on: phonological awareness, alphabetic knowledge, fluency, vocabulary, and comprehension activities. In addition, the observation instrument includes other reading teaching practices based on the use of resources, reinforcement, feedback, modeling, guided oral instruction, homework, reading and writing, and psychomotricity activities.

### Procedure

Before the recordings were made, authorization was obtained from both the teachers and the pupils' parents. All participants provided a written informed consent prior to their participation. The dates and times of the recording sessions were scheduled in advance (taking into account the school timetable). On the first days, the cameras were tested to ensure they were being used properly. Afterward, the cameras were set up in the classrooms 10 minutes prior to the start of the agreed session.

Two cameras and their stands were used to record each teacher. One camera was set up at the back of the classroom, with a full view of the space, to record all instances of teacher-student and student-student interaction. Another camera was placed near the teacher's desk, to record teacher-student interaction and offer a more detailed observation of the teaching. A total of 10 hours of recordings were made for each teacher (1 hour a day, twice a week) in December 2011 and January 2012. Overall, 42 sessions were used in this study.

Over the course of this process, two observers received four training sessions in the use of Match Vision Studio Premium (Perea et al., 2006). Once the training was completed, each observer viewed the same sessions on different occasions (with 15 days in between viewings), so that both intra- and inter-rater reliability could be calculated.

### Data analysis

Data quality was analyzed with Generalizability Theory (Cronbach et al., 1972) to calculate inter- and intra-rater reliability, and the validity of the instrument used. A measurement plan was also developed to calculate the optimal number of sessions required to run the study.

For the measurement plan, the results showed that the absolute and relative generalizability measures were acceptable (at 0.970 and 0.989) at 30 sessions, and that 40 sessions were needed to reach 0.977 and 0.992, respectively. In this sense, a total of 42 sessions were used to have the same number of sessions per teacher.

Regarding inter- and intra-observer reliability, a four-faceted SRC/O (Session, Criterion, Category/Observer) design was used, and analysis showed the greatest percentage of variability to be related to the Criterion facet (33%), while the Observer facet showed no variability at all. The absolute generalizability coefficient was 0.999, and the relative coefficient was also 0.999, showing a high inter-rater reliability.

With respect to the intra-rater reliability, using a four-faceted SRC/M (Session, Criterion, Category/Moment) design, analysis showed that 32% of variability corresponded to the Session facet and 33% corresponded to Criterion, while Moment showed no variability. The absolute and relative generalizability coefficients obtained for Observer 1 were both 0.999. The absolute and relative coefficients for Observer 2 were both 0.997, showing high intra-rater reliability too.

Analyses of validity showed low measures of both absolute (0.000) and relative (0.000) generalizability, which is a clear sign that the test meets specificity criteria.

Next, we analyzed the frequency and duration of the behaviors exhibited by the teachers participating in the study. To determine whether their practices were in line with what the research recommends, we analyzed the frequency and duration of each teacher's use of dimensions mentioned above.

Finally, the T-patterns were analyzed to study the instructional sequence for each teacher. T-pattern detection is used to identify hidden patterns within sequential datasets (Magnusson, 1996, 2000, 2005; Magnusson et al., 2015), and in several fields (Brill et al., 2015; Burgoon et al., 2015; Castañer et al., 2015). A temporal pattern (T-pattern) is essentially a combination of events that occur in the same order with temporal distances between each other that remain relatively invariant in relation to the null hypothesis that each component is independent and is randomly distributed over time. The basic premise here is that the interactive flow or chain of behavior is governed by structures of variable stability that can be visualized by detecting these underlying T-patterns. We considered patterns that had a minimum occurrence of 7 and *p* < 0.05.

## Results

The results showed that the practice used most was feedback, followed by the use of resources, fluency activities, and comprehension previous knowledge activities. Used to a lesser extent were reading-writing activities, (tangible or verbal) reinforcement of correct performance of exercises and reading, alphabetic knowledge activities such as: teaching sounds, letter names, and rules using a visual aid (see Table 2).

**Table 2:** Data on the frequency and duration of teacher's reading practices.

**PRACTICES**		**TEACHERS**					
		**A (%)**	**B (%)**	**C (%)**	**D (%)**	**E (%)**	**F (%)**
Alphabetic knowledge activities	FR	5.27	9.62	9.87	0	0.08	3.21
	DT	3.43	8.08	5.61	0	0.05	1.39
Phonological awareness activities	FR	8.37	5.24	2.16	0	0	3.21
	DT	10.53	6.48	2.57	0	0	5.44
Use of resources	FR	10.66	6.82	8.83	20.05	8.79	4.59
	DT	15.60	5.09	18.34	18.46	8.13	3.11
Comprehension previous knowledge activities	FR	6.19	2.15	1.12	4.36	7.43	1.15
	DT	10.58	1.30	10.96	0.81	4.15	2.76
Reinforcement	FR	7.45	13.74	10.56	11.34	3.41	10.55
	DT	3.13	6.14	3.77	2.20	0.84	2.42
Feedback	FR	29.24	32.66	50.82	22.96	37.57	35.09
	DT	23.50	47.60	42.63	29.53	13.41	29.36
Modeling	FR	0	0.71	0.08	2.32	0	0.69
	DT	0	1.97	0.43	4.13	0	0.97
Fluency Activities	FR	15.25	10.69	6.23	10.17	15.54	24.77
	DT	12.36	7.71	5.93	9.76	27.58	15.04
Guided oral instruction	FR	0.80	0.21	0.08	1.45	0.68	0
	DT	3.37	0.41	0.40	1.96	5.46	0
Homework	FR	0	2.30	0.08	0.58	0.08	2.06
	DT	0	2.44	0.11	0.08	0.08	1.23
Reading and writing activities	FR	8.37	13.57	5.37	4.94	0.25	6.19
	DT	6.61	7.27	5.33	6.93	0.42	25.46
Psychomotricity activities	FR	2.17	0.57	0.60	0.29	0.34	1.37
	DT	3.44	2.30	0.16	0.95	0.55	1.46
Literal or inference comprehension activities	FR	3.78	0.007	3.98	17.73	21.77	5.04
	DT	2.91	0.08	3.56	20.83	34.63	10
Vocabulary activities	FR	2.40	1.72	0	3.77	4.01	2.06
	DT	4.49	3.04	0	4.34	4.05	1.34
Total	FR	99.95	99.91	99.78	99.96	99.95	99.98
	DT	99.95	99.95	99.80	99.98	99.35	99.98

*FR, frequency; DT, duration*.

**Figure 1:**
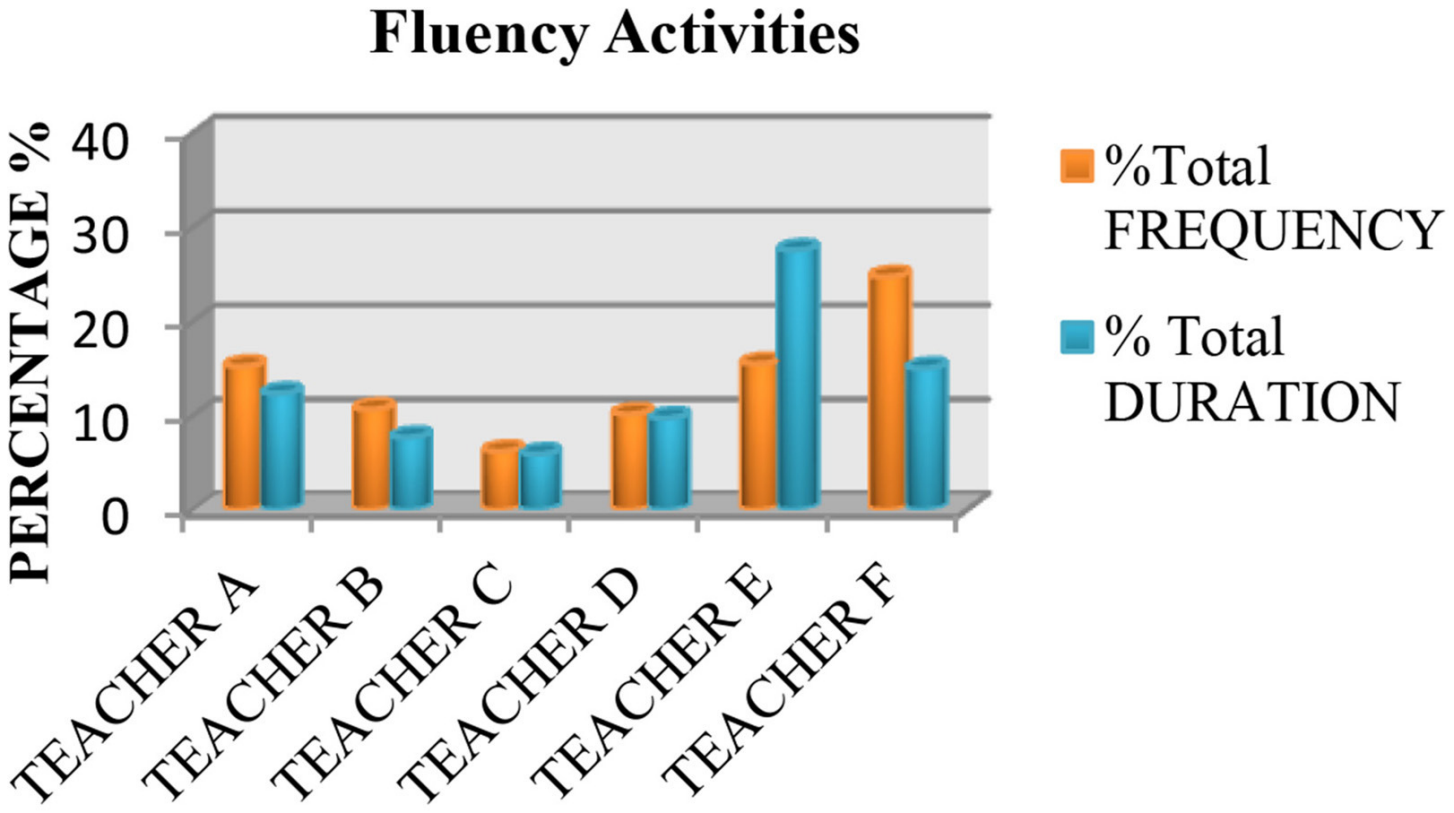
Chart showing the percentage, frequency and duration of six teacher's practice in fluency activities.

**Figure 2:**
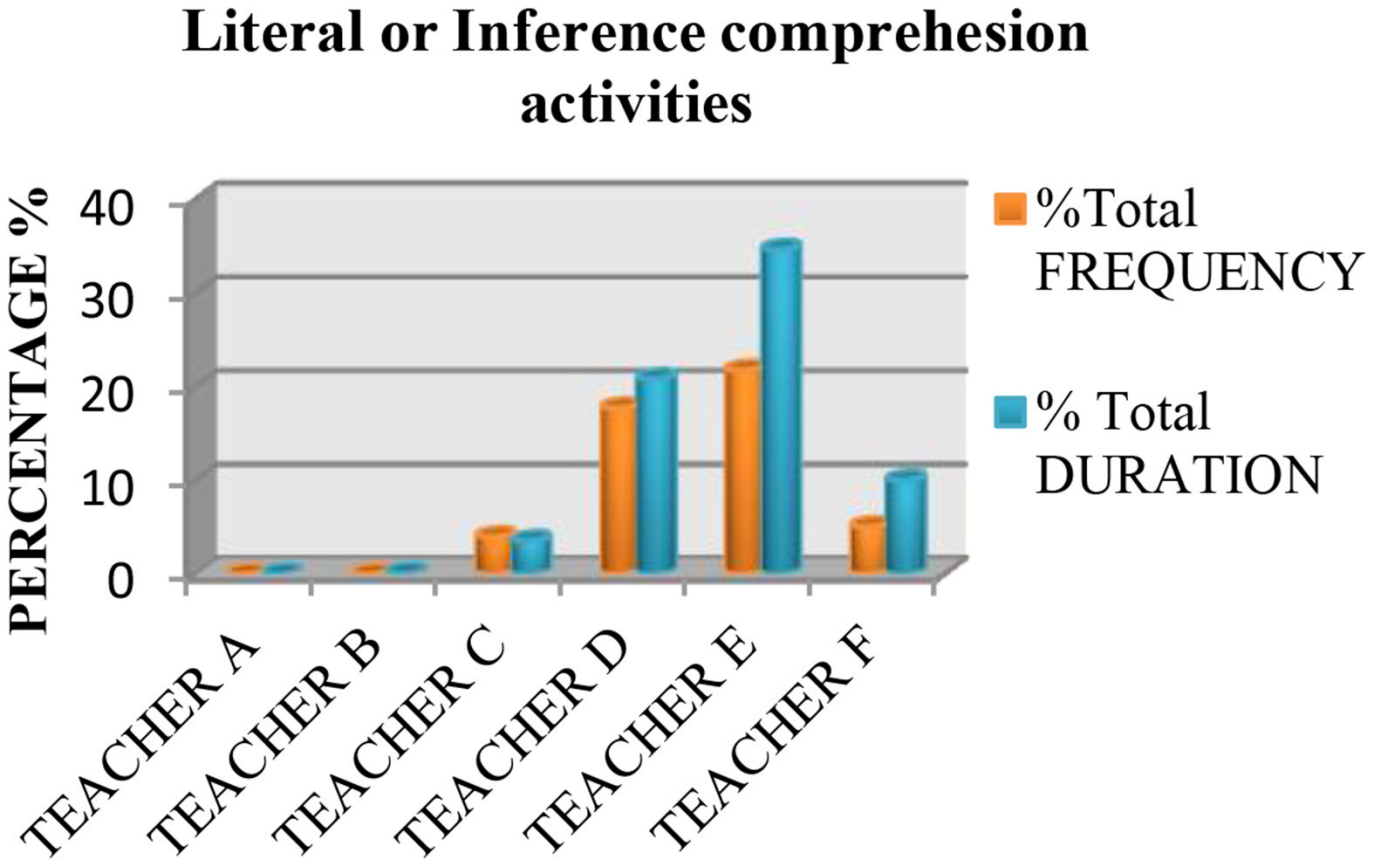
Chart showing the percentage, frequency and duration of six teacher's practice in teaching of functional knowledge of reading.

**Figure 3:**
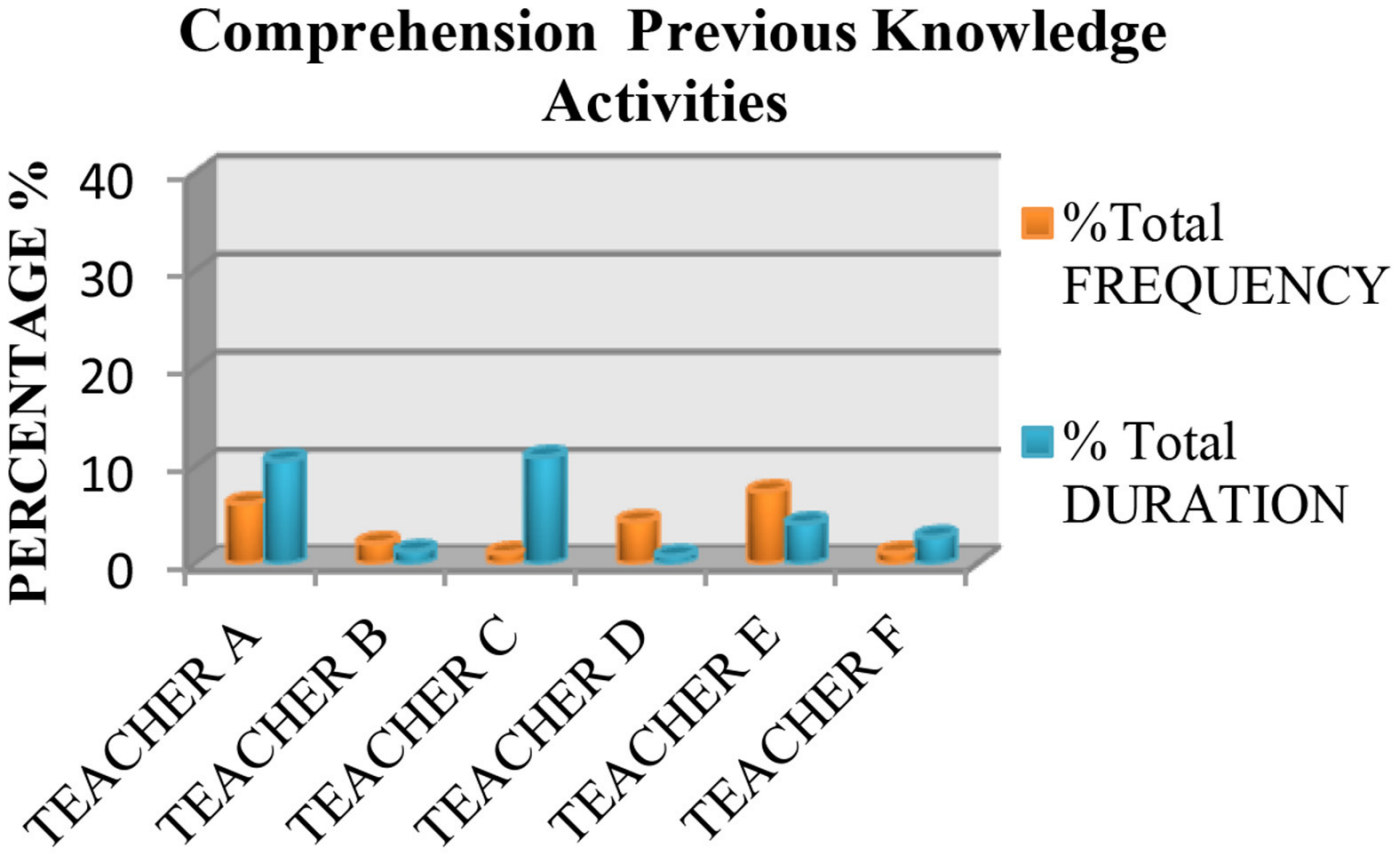
Chart showing the percentage, frequency and duration of six teacher's practice in comprehension activities.

**Figure 4:**
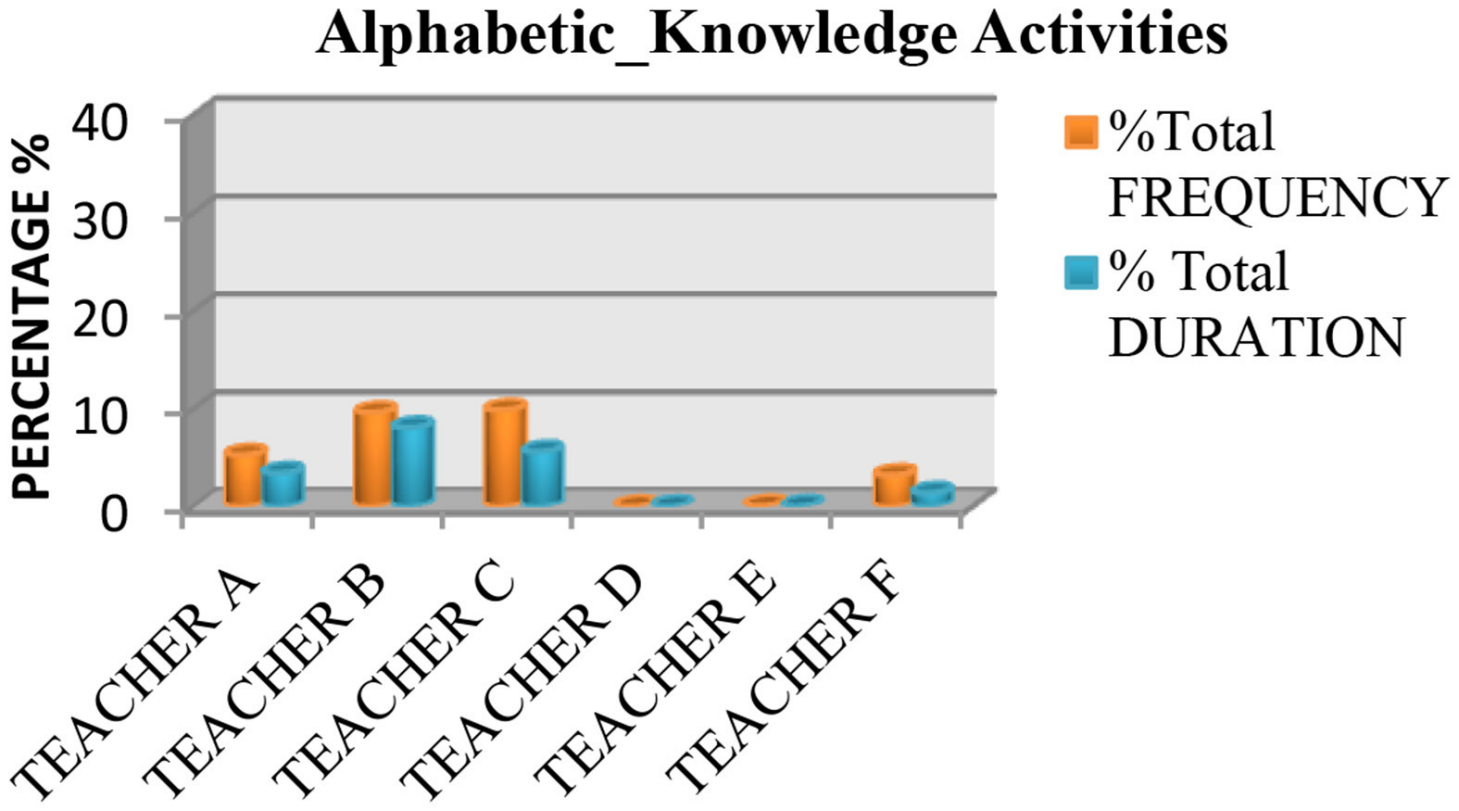
Chart showing the percentage, frequency and duration of six teacher's practice in alphabetic knowledge activities.

**Figure 5:**
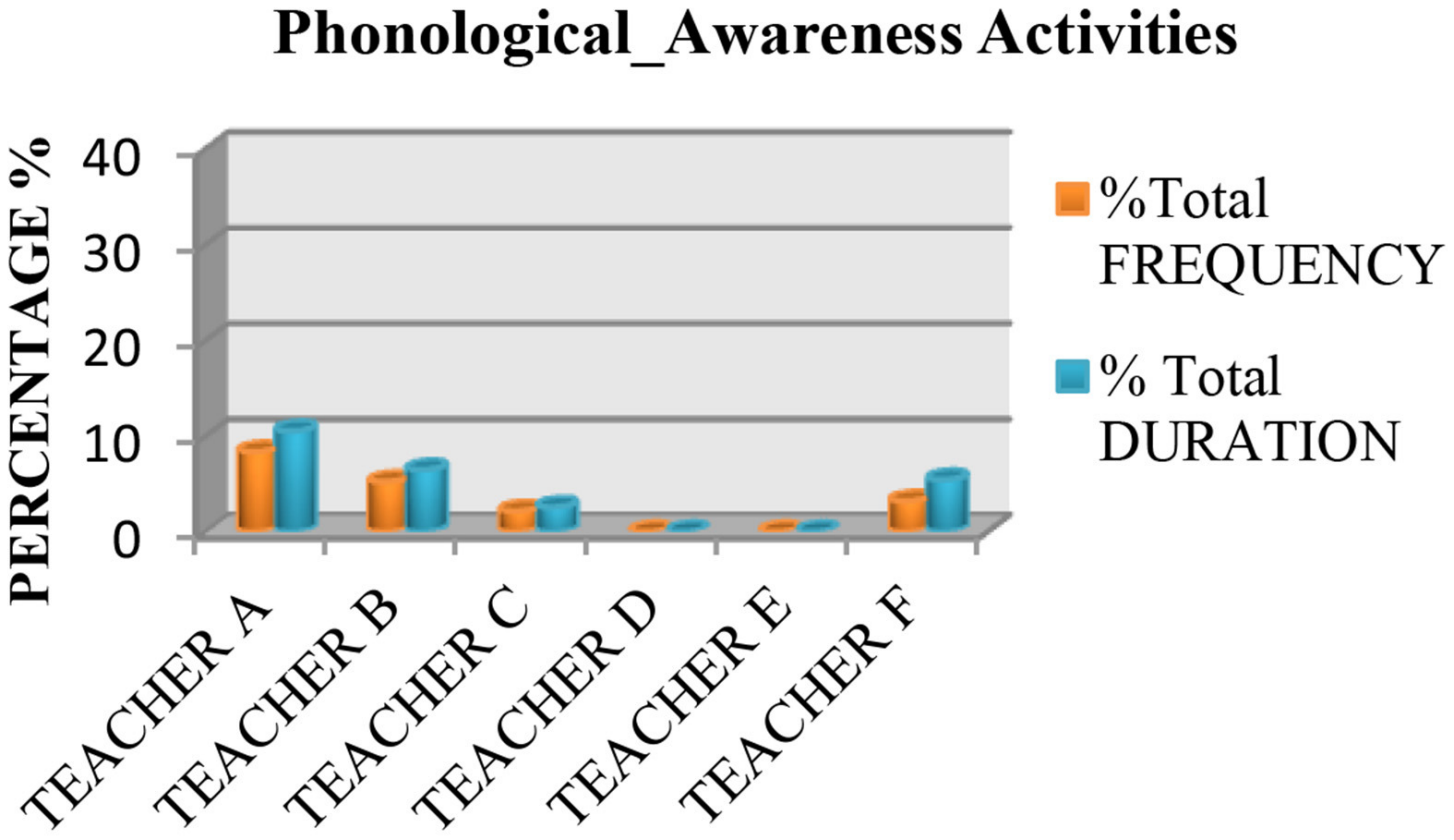
Chart showing the percentage, frequency and duration of six teacher's practice in phonological awareness activities.

**Figure 6:**
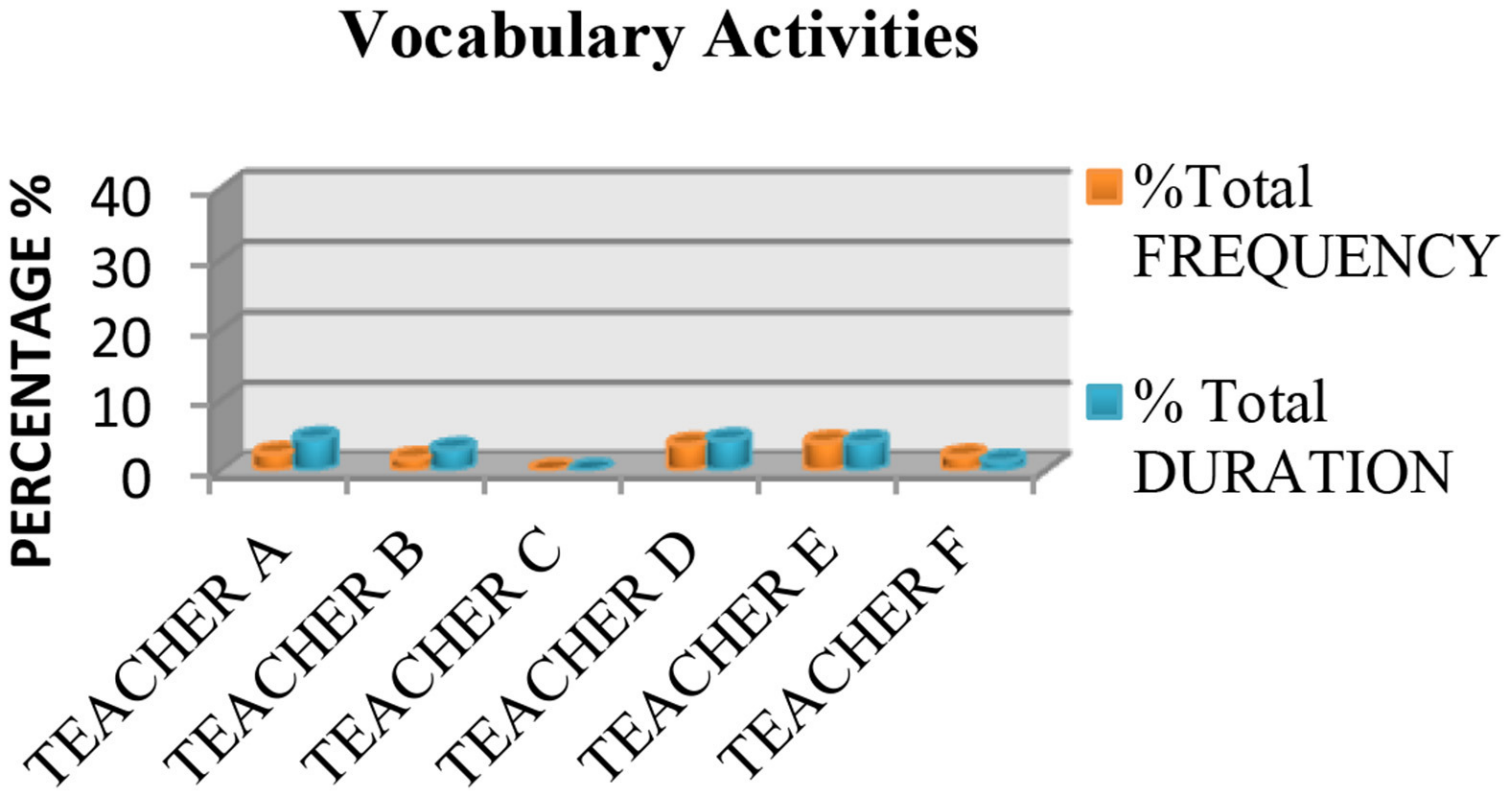
Chart showing the percentage, frequency and duration of six teacher's practice in vocabulary activities.

We also saw that none of the teachers used practices based on the recommendations of the National Reading Panel (2000) more than 50% of the time. Measured in terms of instruction time, all the teachers spent <50% of their time teaching the five essential components of reading, with the exception of Teacher E, who spent 70.46% of class time teaching these components. The data also showed that the most common practice was fluency (see Figure 1), followed by literal or inference comprehension activities (see Figure 2), and comprehension previous knowledge activities (see Figure 3). We also found that teaching alphabetic knowledge activities (see Figure 4), phonological awareness activities (see Figure 5) and vocabulary activities (see Figure 6) were the components addressed the least in class.

**Figure 7:**
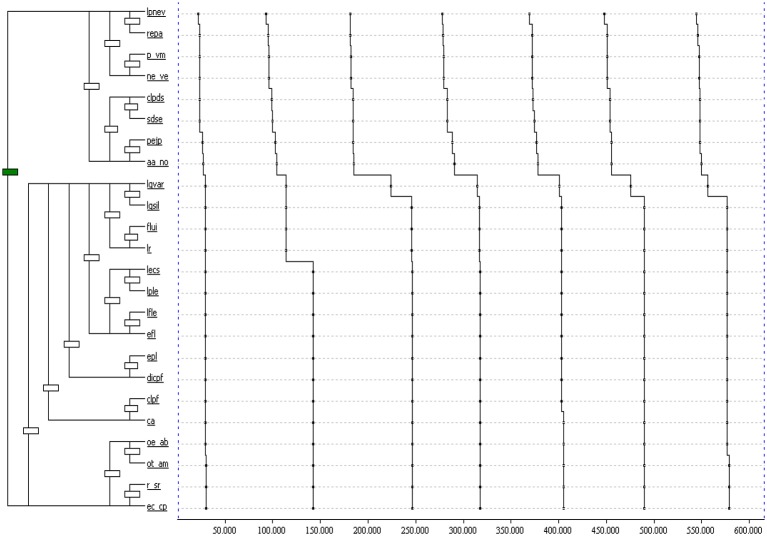
T-patterns. Distribution of instruction sequence for teacher E. You can see the meaning of acronyms in the observation instrument of practices used for teaching reading (Table 1).

To observe whether these practices formed part of a work routine, we analyzed the T-patterns of the six teachers. The results showed that Teacher A was constantly working with comprehension and vocabulary, but that his activities focused exclusively on asking questions and teaching the meaning of words. Teacher B's work routine was based on using activities for developing the five essential components of reading. Thus, in this classroom we observed instruction based on teaching fluency through activities such as rapid, accurate, and precise reading and individual/group reading, as well as joint comprehension and vocabulary work in the form of activities such as relating illustrations to text, doing exercises from the book, asking questions, and studying the meanings of words. Also typical for this teacher's work was running many different activities for teaching phonological awareness and alphabetic knowledge. Teacher C worked first on alphabetic knowledge and then on phonological awareness. No other best practices were observed in that teacher's classroom. Teacher D taught comprehension and vocabulary, but did not demonstrate any appropriate practices related to developing fluency, phonological awareness or alphabetic knowledge. Teacher E's work routine was focused on activities involving rapid, fluent, and accurate reading as well as individual/group and silent reading. There were no other best practices identified in this teacher's sequence of instruction (see Figure 7). The selection of this pattern is due to the fact that it is the teacher who uses most of his time to teach evidence-based components. If we analyze the results, we observe a stable T-pattern over time. The T-Patterns are plotted as dendrograms, the interpretation is performed from top to bottom, and beginning with the most elementary levels of the dendrogram. The T-pattern that is analyzed, consists of two dendrograms. The first one indicates that Teacher E works comprehension by asking the children to relate their experiences (LPNEV) and reviewing the contents worked in the classroom in relation to reading (REPA). Later, he uses positive reinforcement (P_VM) and negative reinforcement (NE_VE). Also, as for the feedback, he corrects the student when he is wrong (CLPDS), he indicates where the error is when reading (SDSE), provides examples (PEJP) and rejects when he is wrong (AA_NO). The second dendrogram indicates that the teacher works fluency through the individual reading aloud and fast (LGVAR), group and silent (LGSIL), and fast reading (LR). In relation to reading and writing, this teacher firstly asks the children to read and then write the word (LPLE) or phrases (LFLE) and vice versa (EPL-EFL). He also uses activities such as dictation (DICPF), copying (CLPF) complete words or phrases (CA). In addition, he instructs with activities that develop psychomotricity, such as orientation in space (OE_AB) or time (OT_AM), rhythm with rhythmic sequences (R_SR) and body schema (EC_CP). Teacher F used practices based on teaching phonological awareness and alphabetic knowledge activities such as: saying words that start with a given sound, dividing words up into syllables, rhyming, and teaching rules using aids. This teacher also worked on fluency activities, asking the children to read out loud using different combinations as well as quickly, accurately, and precisely. No activities aimed at developing vocabulary and comprehensions were observed in this classroom.

We can conclude that no teacher followed a sequence of instruction that was based on teaching all of the components recommended in the scientific literature. Three teachers did not consistently work on vocabulary or comprehension. Three did not include activities for working on phonological awareness or alphabetic knowledge in their practice. We also found that some of the activities run for some of the components were insufficient. For instance, there was no use in certain cases of practices involving isolation, identification, or deletion, and in some cases the teachers even confused phonological awareness with alphabetic knowledge: our observations included situations where the teachers were teaching this skill with alphabet cards hanging on the wall for all the pupils to see, even though they were only meant to be teaching the sounds. For alphabetic knowledge, the activities focused on teaching the name of the letter, the rule, and rhymes.

## Discussion

The case studies presented here through observational methodology have allowed us to analyze if the reading teaching practices used by the teachers in the classroom context are evidence based. That is, we have tried to investigate if these practices promote the skills prescribed by the NRP (i.e., alphabetic knowledge, vocabulary, fluency, comprehension, phonological awareness).

Our findings showed that none of our teachers used practices based on the recommendations of the National Reading Panel (2000) more than 50% of the time. What is more, the T-pattern analysis showed that no teacher studied, had an instruction sequence that was based on some of the key components. The practice that was used the most was feedback, followed by the use of resources, fluency activities, and previous knowledge comprehension activities. To a lesser extent, we saw the use of reading-writing activities, reinforcement aimed at providing (tangible or verbal) praise, reading and writing, and alphabetic knowledge activities. In one of the few studies conducted in this field, Tolchinsky and y Ríos (2009) found that teachers used explicit, early, systematic teaching. In another study, in which Barragán and Medina (2008) observed practices in six preschool classrooms, the results showed that practices differ as a function of how the classroom is organized and what material is available. Also, Ríos et al. (2010), working with two third and fourth grade teachers, identified two profiles of practice types: situational (e.g., working on the basis of situations that arise in the classroom, using newspapers, letters, etc.) and instructional (e.g., teaching letter names, linking letters with sounds). Also worth mentioning is the work by Fons-Esteve and Buisán-Serradell (2012), who used natural observation and systematic recording to analyze the practices of 71 preschool and elementary school teachers. Their results showed that 39% of these teachers used instructional practices, 18% used multidimensional practices, and 14% used situational practices. Looking at all this research, we see that the main strategies analyzed focused on the instructional characteristics, and classified practices as instructional, situational or multidimensional and in terms of the available resources.

However, a common denominator that we observed in the abovementioned studies and which we present here was a far cry from the activities recommended by the National Reading Panel (2000), which insists, for instance, on the need to teach alphabetic knowledge through methods that are synthetic (converting letters to sounds, mixing sounds to form words), analytic (identifying words and their sounds), spelling-based (transforming sounds into letters), contextual (using sound-letter correspondence and finding unknown words in a text), and analogical (using parts of written words to find new ones). With respect to teaching vocabulary, we only observed practices related to teaching the meaning of words and using the dictionary. It has been recommended that when teaching this component, new technologies should also be used (Ito, 2009; Smeets et al., 2014; Bus et al., 2015), as well as the indirect method and repeated exposure to words and their meaning (Daniels, 1994, 1996; Dole et al., 1995); also, this component should be taught early on to promote reading success and comprehension (Joshi, 2005). With respect to comprehension, only three teachers carried out activities of this type, such as linking an illustration with a text, asking questions or summarizing. These exercises should be complemented with monitoring comprehension, cooperative learning, the use of graphic and semantic organizers and recognizing story structure, all of which are activities that have been shown to predict reading success (National Reading Panel, 2000). These results are in line with those obtained in other studies (Moats and Foorman, 2003; Foorman and Moats, 2004; Moats, 2009), where it was found that teachers were not using evidence-based practices.

One alternative would be to promote professional development among teachers to help them keep their knowledge up-to-date. We are aware that participation rates in this type of training are low, as evidenced by the data obtained through the Progress in International Reading Literacy Study (PIRLS) (Mullis et al., 2007); which showed that teachers in Spain receive less training in teaching reading than their counterparts in Bulgaria or Lithuania. The fact is that teacher quality predicts pupils' academic success (European Commission, 2008). Teachers should therefore be given the tools they need to teach properly, using research-based practices. Training programs should therefore address both the fundamentals of theory and educational research on the development and structure of language and reading; offer effective strategies and materials for teaching reading and writing; teach techniques for evaluating a pupil's reading performance as measured by the different components; expose teachers to new technologies; and help teachers strike the right balance between theory and practice (IRA, 2007). A clear example of this can be found in the DIPELEC (*Diploma de Especialización en Enseñanza de la Lectura*), the first postgraduate diploma in reading instruction to be offered in Spain (http://fg.ull.es/grados-posgrados/estudios/diploma-de-especializacion-en-ensenanza-de-la-lectura/). The difficulty now lies in convincing teachers of the need to obtain up-to-date training and change their consolidated teaching practices. Including best practices in legislation and offering compensation to teachers might serve as a good start. A limitation of this study was not analyze how these practices could influence reading performance amongst schoolchildren. Future lines of research should explore this aspect.

## Author contributions

NS: This author's grant was used to run the project *Integrando creencias y prácticas de enseñanza de la lectura* (Integrating beliefs and practices about teaching reading), ref: PSI2009-11662. She participated actively in the observation of the teachers, carried out the analyses of the teaching practices, and was responsible for the literature review and the drafting of this manuscript. CS: Supervised the design and preparation of the study, was responsible for handling and analyzing the data, offered guidance on methodology, and helped review the manuscript. JJ: As principal investigator, supervised the project and the preparation of the study, offered guidance for the theoretical component, and was responsible for reviewing this manuscript. MTA: Carried out the analyses of the teaching practices using T-patterns, offered guidance on methodology, and helped review this manuscript. All authors approved the final version of this article.

### Conflict of interest statement

The authors declare that the research was conducted in the absence of any commercial or financial relationships that could be construed as a potential conflict of interest.
